# Health economic modeling to assess short-term costs of maternal overweight, gestational diabetes, and related macrosomia – a pilot evaluation

**DOI:** 10.3389/fphar.2015.00103

**Published:** 2015-05-20

**Authors:** Irene Lenoir-Wijnkoop, Eline M. van der Beek, Johan Garssen, Mark J. C. Nuijten, Ricardo D. Uauy

**Affiliations:** ^1^Department of Pharmaceutical Sciences, Utrecht UniversityUtrecht, Netherlands; ^2^Danone Nutricia Early Life NutritionBiopolis, Singapore; ^3^Nutricia Research, UtrechtNetherlands; ^4^Ars Accessus Medica, AmsterdamNetherlands; ^5^Institute of Nutrition INTA, University of ChileSantiago, Chile

**Keywords:** maternal overweight, gestational diabetes mellitus, macrosomia, health economics, public health, burden

## Abstract

**Background:** Despite the interest in the impact of overweight and obesity on public health, little is known about the social and economic impact of being born large for gestational age or macrosomic. Both conditions are related to maternal obesity and/or gestational diabetes mellitus (GDM) and associated with increased morbidity for mother and child in the perinatal period. Poorly controlled diabetes during pregnancy, pre- pregnancy maternal obesity and/or excessive maternal weight gain during pregnancy are associated with intermittent periods of fetal exposure to hyperglycemia and subsequent hyperinsulinemia, leading to increased birth weight (e.g., macrosomia), body adiposity, and glycogen storage in the liver. Macrosomia is associated with an increased risk of developing obesity and type 2 diabetes mellitus later in life.

**Objective:** Provide insight in the short-term health-economic impact of maternal overweight, GDM, and related macrosomia. To this end, a health economic framework was designed. This pilot study also aims to encourage further health technology assessments, based on country- and population-specific data.

**Results:** The estimation of the direct health-economic burden of maternal overweight, GDM and related macrosomia indicates that associated healthcare expenditures are substantial. The calculation of a budget impact of GDM, based on a conservative approach of our model, using USA costing data, indicates an annual cost of more than $1,8 billion without taking into account long-term consequences.

**Conclusion:** Although overweight and obesity are a recognized concern worldwide, less attention has been given to the health economic consequences of these conditions in women of child-bearing age and their offspring. The presented outcomes underline the need for preventive management strategies and public health interventions on life style, diet and physical activity. Also, the predisposition in people of Asian ethnicity to develop diabetes emphasizes the urgent need to collect more country-specific data on the incidence of macrosomic births and health outcomes. In addition, it would be of interest to further explore the long-term health economic consequences of macrosomia and related risk factors.

## Introduction

The foundations of health throughout life are laid during the peri-conceptional period, from conception until birth, and after birth in early childhood. Much attention has been paid to the long-term consequences of undernutrition and micronutrient deficiencies during the first 1000 days, covering the timespan from conception until the second birthday (Morton, 2006; Black et al., 2008). The link between compromised nutritional status of the baby’s mother and low birth weight on one hand, and impaired health of the child in later life on the other hand has now been clearly established. The far-reaching relationships with multiple health-related outcomes affecting human capital and productivity have been clearly corroborated (Johnson and Schoeni, 2011).

In contrast, despite the general high interest in the public health burden of overweight and obesity, far less is known about the potential clinical and economic consequences of maternal conditions leading to high birth weight (large for gestational age; LGA) or macrosomia.

### Macrosomia

Macrosomia is defined as an absolute birth weight >4000 g regardless of gestational age (Boulet et al., 2003; Costa et al., 2012). The incidence of macrosomia ranges from 12.8 to 37.4% worldwide (Rodrigues et al., 2000; Kac and Velasquez-Melendez, 2005; de Oliveira et al., 2008)^.^ In developed countries, the prevalence of macrosomia ranges from 5 to 20%; and an increase of 15–25% has been reported over the last three decades, mainly driven by an increase in maternal obesity and type 2 diabetes (T2DM). In addition, the threshold for macrosomia might need to be reconsidered for Asian countries, where average birth weight is in general lower compared to European countries and consequently the cut off weight for LGA (>95th percentile) would be lower.

Maternal overweight, excessive gestational weight gain (GWG) by itself, gestational diabetes mellitus (GDM), defined as mild to moderate hyperglycemia leading to diabetes first diagnosed during pregnancy which disappears after giving birth, and elevated fasting plasma glucose levels during pregnancy have all been reported to be significant risk factors for macrosomia (Shi et al., 2014). In developing countries maternal short statue, high body mass index (BMI), and T2DM are strong risk factors for macrosomia (Koyanagi et al., 2013).

Macrosomia is the main cause of (acute) perinatal complications for both mother and infant. Adverse maternal outcomes associated with macrosomia include preterm birth, higher rates of postpartum hemorrhage, as well as increased risk of cesarean delivery (HAPO Study Cooperative Research Group et al., 2008; Henriksen, 2008; Jastrow et al., 2010). For the macrosomic infant, birth trauma is commonly related to instrumental delivery, e.g., newborns with a birth weight >4000 g have 9.0 times higher odds of shoulder dystocia, while those with a birth weight >4500 g have odds that are 39.5 times higher than normal-weight infants (Robinson et al., 2003). Furthermore, macrosomic infants are more likely to have low 5-min Apgar scores, an index of hypoxia (Johnson and Schoeni, 2011). Infants with very severe macrosomia (birth weight >5000 g) are at increased risk of neonatal, post-neonatal and infant death (Boulet et al., 2003). Macrosomia also significantly increases the risk for developing obesity in childhood, and non-communicable diseases (NCD) later in life (Morton, 2006).

## Background

A key component of normal metabolic adaptation to pregnancy is the development of mild insulin resistance and changes in the regulation of appetite in the mother, gradually evolving during gestation (Parsons et al., 1992; Kawai and Kishi, 1999; Clapp, 2006). These normal physiological adaptations serve to shuttle sufficient nutrients to the growing fetus, especially during the last trimester of pregnancy. Poorly controlled diabetes, maternal obesity, and excessive maternal weight gain during pregnancy are associated with intermittent, non-physiological periods of fetal hyperglycemia, and subsequent hyperinsulinemia from the start of pregnancy and onward. The resulting maternal insulin resistance and hormonal responses related to high blood glucose, such as insulin-like growth factors, and growth hormone, lead to greater deposition of body fat and glycogen in muscle and liver in the fetus. The greater and more rapid fetal growth (in particular of adipose tissue) subsequently results in increased birth weight.

### Overweight, Obesity, and Gestational Weight Gain

Women with either pre-pregnancy obesity and/or excessive GWG, have a higher risk for developing GDM, pregnancy-induced hypertension, cesarean delivery, and LGA and macrosomic infants compared to women with normal pre-pregnancy BMI and adequate pregnancy weight gain (Li et al., 2013).

Using a hospital-based delivery database of 18 362 subjects in the USA, overweight, obese and severely obese women showed higher risks for LGA, GDM, and preeclampsia in comparison to their normal-weight counterparts (Bodnar et al., 2010). In another study, the proportion of LGA infants born to overweight and obese mothers without GDM was significantly higher than in their normal-weight counterparts in a retrospective study of 9 835 women in Southern California, USA; 21.6% of LGA infants were explained by maternal overweight and obesity (Black et al., 2013). Similarly, a 13-years study of 292 568 singleton pregnancies in China (Liu et al., 2012) demonstrated that adverse pregnancy outcomes, such as hypertensive disorders, cesarean delivery, macrosomia, and LGA infants, were associated with overweight mothers, who during pregnancy gained weight beyond current IOM recommendations (Institute of Medicine/National Research Council, 2009).

In a study of 366 886 singleton pregnancies from the Danish Medical Birth Registry from 2004 to 2010, the ratio between abdominal circumference and birth weight decreased with increasing maternal BMI, suggesting that maternal obesity results in a general weight gain of the fetus rather than just fat accumulation around the abdomen (Tanvig et al., 2013). Finally, an observational study at five antenatal centers in Ireland reported that excessive GWG resulted in higher odds for LGA and macrosomia, as well as increased odds for gestational hypertension in women with GDM. The need for treatment with insulin further increased the odds for LGA and macrosomia (Egan et al., 2014).

Altogether, these studies emphasize that high pre-pregnancy BMI and/or high GWG form a substantial risk for macrosomic birth worldwide. The fact that some studies do not report increased rates of macrosomia despite the increasing prevalence of obese pregnancies, may be explained by, for instance, changes in obstetric practice such as cesarean section before weeks 40 of pregnancy (Poston et al., 2011).

### Gestational Diabetes Mellitus

In women already prone to insulin resistance because of obesity or (epi) genetic predisposition (Vaag et al., 2014), this physiological tendency is augmented and can result in the development of GDM, commonly diagnosed around weeks 20–24 of pregnancy. A study including 35 253 pregnancies in Australia showed an average incidence of GDM of 5.5% (*n* = 1928; Beischer et al., 1991).

GDM has been reported to affect 4–7% of pregnancies in Caucasian women, while the incidence is consistently higher (8–15%), and rising rapidly in Asian women (Ferrara et al., 2004; Rosenberg et al., 2005; Hunsberger et al., 2010). According to a recent survey, there is a large variation in estimated GDM prevalence, showing a range from <1 to 28% with data derived from single or multi-site, national data, and/or estimates from expert assessments in 47 countries (Jiwani et al., 2012). Direct comparison between countries is difficult due to different diagnostic strategies and population groups. Many countries do not perform systematic screening for GDM, and practices often diverge from guidelines. Interestingly, the hyperglycemia and pregnancy outcome (HAPO) study results clearly indicate that relatively mild hyperglycemia was already associated with a significant increase in macrosomia (Zawiejska et al., 2014). Adoption of the HAPO criteria for GDM diagnosis will likely lead to higher GDM prevalence compared to current estimates (Jiwani et al., 2012), although still considerable differences in incidence as well as relevance of the different hyperglycemia measures were reported between the participating HAPO centers (Sacks et al., 2012).

## Objective

The primary objective of this study was to design a health economic framework that will allow a pilot estimation of the short-term healthcare burden associated with maternal overweight and/or GDM, in particular as related to fetal macrosomia. The secondary goal is to lay a basis for fostering interest in the development of targeted preventive approaches in an effort to reduce the related total costs. The subject is closely related to the problem of rising NCD prevalence and the related disease outcomes, and will be of interest for both developing and industrialized countries (Henriksen, 2008; Ma and Chan, 2013).

## Materials and Methods

A model to map the health economic consequences of GDM, overweight pregnancies and macrosomia was developed based on decision analytical techniques, a well-accepted methodology in the field of health-economics (Weinstein and Fineberg, 1980). To estimate the health economic impact of management of macrosomia, the short-term consequences of GDM, obesity and macrosomia were taken into account. Data sources included published literature, clinical trials, official price/tariff lists, if available, and national population statistics. This study is based on methodological guidance derived from cost-effectiveness studies in nutrition economics (Lenoir-Wijnkoop et al., 2011).

### Model Design

**FIGURE 1:**
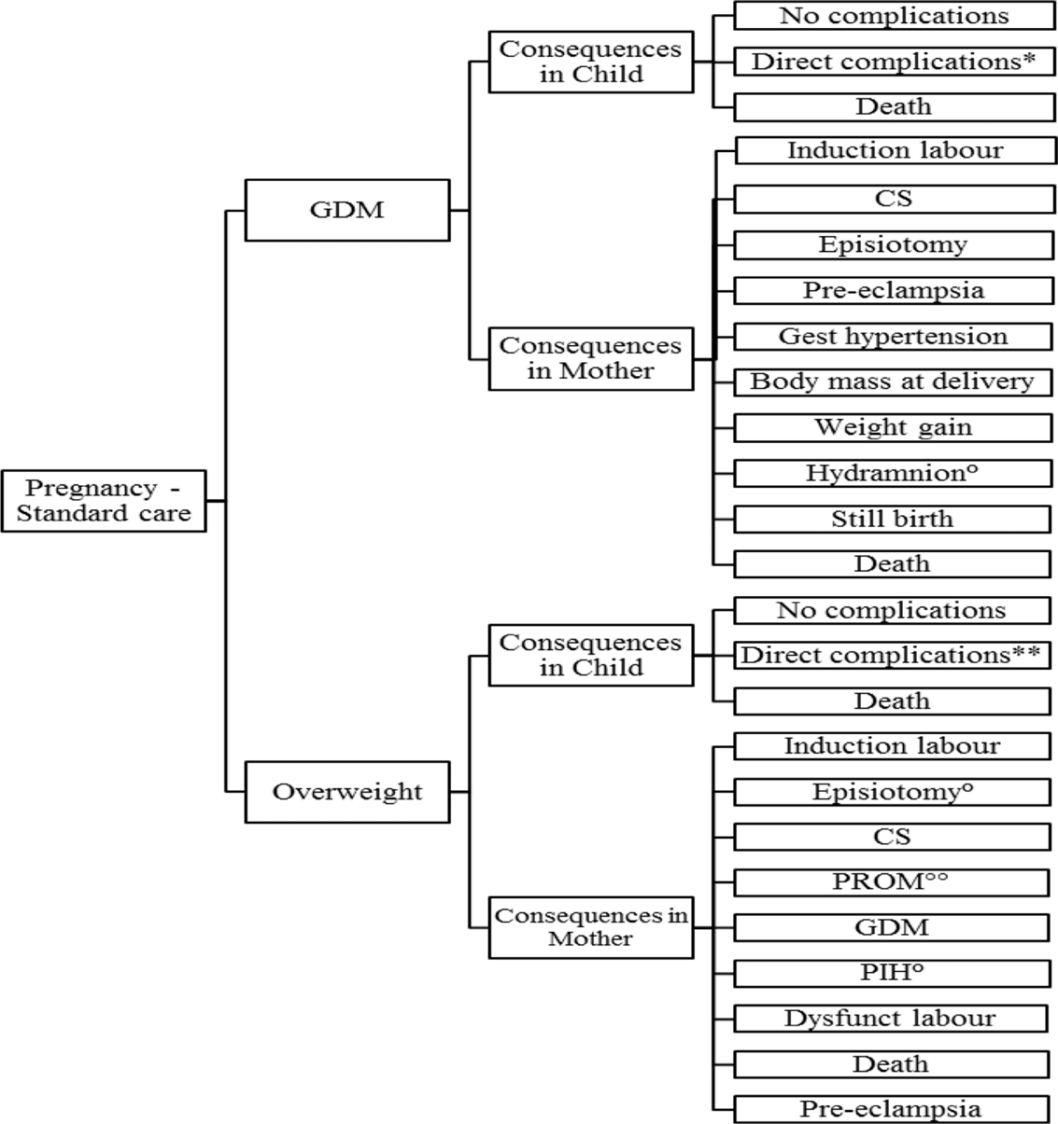
**Structure of the model.** Reported events are based on literature: ^∗^Includes respectively, hypoglycemia, hyperbilirubinemia, prematurity, macrosomia, brachial plexus injury, shoulder dystocia, respiratory distress, NICU admission; ^∗∗^Includes respectively, prematurity, macrosomia, NICU, stillbirth, IUGR; °Only reported for GDM (Michlin); ^∘∘^Not reported for GDM.

The health economic impact is calculated, using a decision tree model constructed in TreeAge Pro 2005/2006, reflecting treatment patterns and outcomes in the management of obesity during pregnancy, GDM and related delivery of the macrosomic infant. The present decision tree model is shown in **Figure 1**.

During the pregnancy the occurrence of GDM and/or obesity may lead to various complications in mother and child. The model consists of two sub-models: (1) development of maternal GDM, (2) maternal obesity, accompanied or not with the development of GDM.

Delivery after GDM or obesity is the next “health state.” The delivery may be normal, leading to “normal child” or “macrosomic” child. Using conventional principles of clinical decision analysis, expected clinical, and economic outcomes are determined as a probability-weighted sum of costs and outcomes further to the initial treatment decisions. As both mother and child may be subject to various clinical events and disease progression after delivery, the number of possible health states is finite. Therefore the follow-up beyond delivery was based on a Markov process. An advantage of applying a Markov process is that it allows long-term modeling of GDM and obesity for the mother and its complications for both mother and child (complicated delivery, macrosomia, and related morbidity).

### Study Population

The model included a study population of women of childbearing age who are overweight or obese prior to pregnancy. Women with (preexisting) diabetes mellitus, both type I and type II, or related morbidity before pregnancy were excluded. The model considers thus a cohort of otherwise healthy women with a probability of getting pregnant.

### Cost Assessment

An analysis can be conducted from the perspective of the society in a pre-selected study country, while it is also possible to consider the payer’s perspective only. The choice of the perspective will depend on the country-specific health economic guidelines. The current cost assessment, performed as a pilot, is based only on short-term costs caused by the management of the complications as reported in literature, from the national health care perspective.

## Data Sources

Various data sources were considered for developing the framework in order to maximize its external validity for any local setting. A narrative review of the scientific literature from several electronic databases was conducted to find studies published between 1994 to July 2014 with the following keywords: birth weight, (rapid) weight gain, growth trajectories, body composition, overweight, obesity, metabolic health, cohort, observational studies, Asia, Australia, and Europe. Only studies published in English were included. Probabilities of clinical events and utilities are usually accepted as not country-specific and are considered to be transferable beyond their original production location. They can therefore be derived from international studies, while economic measures and information on therapeutic choices depend on a particular region, country or healthcare system (Lampe et al., 2009).

### Incidence

The incidence rate used for our model was 5.5%, derived from the study outcomes reported by Beischer et al. (1991). This is a conservative value, taken into account the incidence rates reported above (Ferrara et al., 2004; Rosenberg et al., 2005; Hunsberger et al., 2010), and considering the rising risk of macrosomic pregnancies related to an overall 15–25% increase in the proportion of women giving birth to large infants worldwide (Henriksen, 2008).

### Complications

Studies reporting the risk of perinatal adverse outcomes for mother and child in the case of obesity (Galtier-Dereure et al., 1995; Michlin et al., 2000; Pathi et al., 2006; Salihu et al., 2011), mild GDM (Landon et al., 2009; Ohno et al., 2011), and total GDM (Keshavarz et al., 2005; Reece, 2010; Mission et al., 2012) show that not all complications are statistically significant. Data input on obesity is derived from Salihu et al. (2011) because of the large sample size of obese women (**Table 1**), whereas for GDM the data for mild GDM are used (Galtier-Dereure et al., 1995; Pathi et al., 2006), taking a conservative approach. Mission et al. (2012) provided a much higher probability for shoulder dystocia, which was taken into account for further cost estimations, as well as additional information provided by Keshavarz et al. (2005) on the probability of hydramnion and stillbirth (respectively, 0.60 and 0.40%).

**Table 1:** Complications in mild gestational diabetes mellitus (GDM) and obese mothers.

Mild GDM								Source
**Outcomes**								**Landon**

**Neonatal outcomes**	**Hypo-glycemia**	**Hyper-bilirubinemia**	**Birth weight >4000**	**Preterm delivery**	**NICU admission**	**Resp. distress**	**Fat mass in g**	

Intervention (*n* = 485)	16.30%	9.60%	5.90%	9.40%	9.00%	1.90%	42,700	
Control (*n* = 473)	15.40%	12.90%	14.30%	11.60%	11.60%	2.90%	46,400	
Statistical significance	NS	NS	S	NS	NS	NS	S	

**Maternal outcomes**		**Induction labor**	**Cesarean delivery**	**Shoulder dystocia**	**Preeclampsia**	**Preeclampsia OR gestational hypertension**	**Body mass at delivery**	**Weight gain (g)**

Intervention (*n* = 485)		27.30%	26.90%	1.50%	2.50%	8.60%	3130	280
Control (n = 473)		26.80%	33.80%	4.00%	5.50%	13.60%	3230	500
Statistical significance		NS	S	S	S	S	S	

**Maternal outcomes^∗^**		**Preeclampsia**	**Cesarean delivery**					**Ohno**

Treatment		8.60%	26.90%					
No treatment		13.60%	33.80%					

**Neonatal outcomes**^∗^		**Macrosomia**	**Brachial plexus injury**	**NICU admission**				

Treatment		5.90%	6.70%	9.00%				
No treatment		14.30%	6.70%	11.6%				

**Obese mothers**								

	**Anemia**	**Insulin-diabetes**	**Other diabetes**	**Chronic hypertension**	**Preeclampsia**	**Eclampsia**		**Salihu**

Non-obese (*n* = 90,022)	1.18%	0.83%	2.18%	0.28%	2.42%	0.06%		
Obese (*n* = 26,954)	1.31%	3.08%	7.18%	2.23%	5.89%	0.08%		
Statistical significance	NS	S	S	S	S	NS		

*Statistical significance not reported.

### Macrosomia Management in GDM

**Table 2** shows an overview of data from studies on interventions related to macrosomia incidence in GDM (Langer et al., 2005; Horvath et al., 2010).

**Table 2:** Treatment GDM – macrosomia.

Risk	Comparison	Odds ratio	Confidence interval	Source
Macrosomia	Treatment GDM vs. usual care	0.38	0.30–0.49	Horvath
Macrosomia	No treatment GDM vs. control	2.66	1.93–3.67	Langer
Macrosomia	Treatment vs. control	1.13	0.82–1.55	Langer
LGA (large for gestational age)	No treatment GDM vs. control	3.28	2.53–3.67	Langer
	Treatment GDM vs. control	1.06	0.81–1.38	Langer

Herbst (2005) provided data on direct complications related to macrosomia. These data may be considered in addition to previously mentioned data. Using decision analysis techniques, the authors compared three strategies for an infant with an estimated fetal weight of 4500 g: labor induction, elective cesarean delivery, and expectant treatment (**Table 3**).

**Table 3:** Complications macrosomia.

	
**Fetal macrosomia**	**Cesarean delivery**	
	Elective induction	35%
	Expectant mgt	33%
		
	**Shoulder dystocia**	
	Elective cesarean delivery	0.1%
		
	Elective induction	
	Ceasarean delivery	0.3%
	Vaginal delivery	14.5%
		
	**Expectant management**	
	Ceasarean delivery	0.3%
	Vaginal delivery	3%
	Plexus injury	18%
	Permanent injury	6.7%

Mortality outcomes were based on the study by Mitanchez (2010) who evaluated the risks of perinatal complications in infants born to mothers with treated or untreated GDM, including also risk of death.

Most of the costing data were derived from the studies by Herbst (2005), Ohno et al. (2011). In case of lack of information on direct data, the costs were based on treatment practice derived from guidelines or assumptions based on similarities in treatment (**Table 4**). Maternal short-term costs are related to cesarean section, pre-eclampsia, or gestational hypertension, induction of labor, maternal death. In this model we assume that in case of normal pregnancy and vaginal delivery, there is a routine cost of $ 7 790 (Ohno et al., 2011). This assumption is, however, based on the 2011 situation in the USA only, and outcomes may be considerably different in case specific costing data of other countries or at other time points would be used. Because of the lack of costing data from other countries, we performed an extreme sensitivity analysis on the costs by varying ±20%.

**Table 4:** Costing data.

Cost item	Cost ($)	Cost item	Cost ($)
Child_brachplexus	1,757	Mother_anemia	0
Child_hyperbili	2,006	Mother_bodymass	0
Child_hypoglycemia	2,419	Mother_cesarean	4,189
Comp_child_IUFD	82,361	Mother_episiotomy	5,165
Mp_child_IUGR	15,065	Mother_gdm	1,786
Child_macrosomia	4,014	Mother_gest	1,786
Child_NICU	15,065	Mother_gesthyper	1,786
Child_overweight	4,014	Mother_hydramnion	0
Child_premature	3,376	Mother_hypertension	1,786
Child_pretermdelivery	3,376	Mother_induction	5,165
Child_resp_distress	3,376	Mother_PIH	19,184
Child_shoulder	1,757	Mother_pre eclampsia	19,184
		Mother_PROM	5,165
		Mother_shoulder	950
		Mother_still birth	0
		Mother_weight gain	0
Assumption routine cost normal pregnancy and vaginal delivery			7,790

## Results

The base case analysis gives the results for the period including pregnancy and delivery only, without including costs of diagnosis and management of GDM, nor of complications beyond the obstetric period or consequences for mother and child on the longer term.

The average of total additional costs for overweight is $ 18 290 per pregnancy/delivery, which consists of average costs for the mother ($ 13 047), and average costs for the child ($ 5 243).

The average of total additional costs for GDM is $ 15 593 per pregnancy/delivery, which consists of the average costs for delivery and complications for the mother ($ 11 794) and the average direct costs for neonatal complications in the macrosomic child ($ 3 799; **Table 5**).

**Table 5:** Base case analysis.

	Mother	Child	Total
Period		Pregnancy and delivery	
Normal	$7,790	$0	$7,790
GDM	$11,794	$3,799	$15,593
Overweight	$13,047	$5,243	$18,290

### Example of a Budget Impact Calculation

The translation of costs per case (pregnancy and delivery only) to national level, based on pregnancy rate and the incidence of GDM, leads to the budget impact. To illustrate this, the budget impact of GDM for the USA was calculated, since most of the costing data available are provided by USA studies. The national annual number of pregnancies is 13.68 per 1000 for a population of 313 847 500 (Indexmundi, 2014; www.indexmundi.com). In case of a GDM incidence rate of 5.5% (Zawiejska et al., 2014), this represents an annual number of GDM cases of 236 139 in the US. With a cost difference between normal pregnancy/delivery and complicated delivery due to GDM of $7 803 ($15 593 – $7 790), this leads to an annual budget impact of more than $1.8 billion, according to the short term conservative approach taken in our model. Although these outcomes cannot be extrapolated to other countries because of differences in costs as well as in the organization of national health structures, the principle of calculation remains similar for any part of the world, and will be of use as soon as reliable information becomes available.

**Table 6** shows an overview of the sensitivity analyses. Because of lack of statistical distributions, the sensitivity analyses were conducted by varying the parameters ±20%. The outcomes show that in all sensitivity analyses the economic impact remains substantial.

**Table 6:** Sensitivity analyses.

	Per case	BIA
Base case	$7,803	$1,842,525,634
**Incidence**		
-20%	$7,803	$1,474,020,507
20%	$7,803	$2,211,030,761
**Cost complications**		
-20%	$4,684	$1,106,116,165
20%	$10,921	$2,578,935,103
**Cost normal pregnancy**		
-20%	$6,242	$9,363
20%	$1,474,020,507	$2,211,030,761
**Cost complications baby**		
-20%	$5,444	$10,161
20%	$1,285,536,028	$2,399,515,240
**Cost complications mother**		
	$7,043	$8,563
	$1,663,105,770	$2,021,945,498

## Discussion

The current model proposes to assess the health economic consequences of macrosomia. Based on international epidemiological and US population costing data, it was shown that the budget impact related to short term obstetric complications for both mother and child is considerable. The presented model offers a first approach for further health technology assessments in different parts of the world and can be used with country specific data to evaluate cost-effectiveness of proposed preventive interventions to reduce the current and future public health consequences of macrosomia. It is anticipated that the reported pilot assessment using available US costing data provides a conservative picture of the true health economic impact of macrosomic births, given the reported increase in maternal overweight and obesity, not only in developed but also in developing countries. The recent debate on diagnostic criteria for GDM stirred by the linear relationship between maternal hyperglycemia and fetal outcomes adds further fuel to this assumption (Jiwani et al., 2012; Sacks et al., 2012).

### Relevance and Applicability of this Framework

Maternal BMI, nutritional status and dietary intake are the main determinants of fetal growth as well as the occurrence of maternal hyperglycemia. The latter may result in GDM, defined as diabetes first diagnosed during pregnancy, and is particularly prevalent –and increasing rapidly– in the Asian regions (Hunsberger et al., 2010). Ethnic differences play a pivotal role in the risk for fetal macrosomia. Worldwide, the rising epidemics of overweight in girls and women of child-bearing age do not bode well and calls for preventive strategies (Mulla et al., 2010).

A limitation of this modeling approach lies in the lack of randomized trial evidence on targeted lifestyle interventions in pregnancy and their effect on birth outcomes (Balaji et al., 2014; Briley et al., 2014). However, as maternal overweight, excessive GWG by itself, GDM, and elevated fasting plasma glucose levels during pregnancy have all been reported to be significant risk factors for macrosomia (Liu et al., 2012; Black et al., 2013; Li et al., 2013; Shi et al., 2014), it seems reasonable to assume that a reduction of GDM (severity) and obese pregnancies would lead to fewer complications and thus decrease the related health care costs. Another limitation of the presented framework is its restriction to short-term costs only. More and more evidence is emerging on the increased long-term risks for macrosomic babies to develop future health concerns, including metabolic syndrome, diabetes, and cancer. Besides the further increase of related health care expenditures, this also raises the question of the impact on the next generations (Catalano, 2003; Roseboom and Watson, 2012), which argues in favor of implementing health strategies that may contribute to prevent a vicious circle of NCD.

Dietary management and exercise are potentially effective interventions to prevent excessive weight gain and GDM if measures are established before or in the early stages of pregnancy (Thangaratinam et al., 2012). Evidence from observational studies and clinical trials indicates that dietary energy intake and the source of energy influences glucose metabolism and insulin responses (Hu et al., 2001; Galgani et al., 2008). High fat diets, likely to be unbalanced in their macronutrient composition, have been demonstrated to increase the risk for GDM recurrence in future pregnancies (Moses et al., 1997). An evaluation of pregnancy management in women with GDM or gestational mild hyperglycemia in France demonstrated that there were no LGA babies in women whose carbohydrate intake was at least 210 g/day (Romon et al., 2001) indicating the significance of sufficient carbohydrate intake during pregnancy. The study suggested that nutrition counseling should be directed at an adequate carbohydrate intake of 250 g/day, while maintaining a low fat diet to limit the total energy intake. Indeed, higher consumption of saturated fat and trans fat as a percentage of total energy intake, added sugar and lower intake of vegetables and fruit fiber during the second trimester of pregnancy were associated with greater risk for glucose intolerance during the last trimester of pregnancy (Ley et al., 2011). A similar study suggests an association between saturated fat and sugar intake during the second trimester with not only birth weight, but also body weight, and adiposity in the offspring at 5 years of age (Murrin et al., 2013). A ‘high’ glycemic diet resulting in elevated postprandial glucose levels compared to a ‘low’ glycemic diet may significantly increase birth weight in healthy pregnant women (McGowan and McAuliffe, 2010; Tzanetakou et al., 2011). Although these studies suggest that a balanced macronutrient intake as well as carbohydrate quality play a crucial role in dietary management of GDM, health economic costs assessment of dietary approaches to date is limited.

### Long-Term Risk of Gestational Diabetes Mellitus

The current pilot analysis focusses only on costs related to perinatal complications of macrosomic birth. Several studies on the association between GDM and long-term risk of diabetes mellitus show that women with GDM also have a greater risk of developing diabetes in the future compared to pregnant women with a normal glucose tolerance (Bellamy et al., 2009; Jiwani et al., 2012).

A review by Henry and Beischer (1991) provides similar results. Using life table techniques, 17 years after the initial diagnosis of GDM, 40% of women were diabetic compared with 10% in a matched control group of women who had normal glucose tolerance in pregnancy. The incidence of diabetes was higher among women who were older, more obese, of greater parity, and with more severe degrees of glucose intolerance during pregnancy. Diabetes also occurred more commonly among women who had a first-degree relative who was diabetic, in women born in Mediterranean and East Asian countries, and in those who had GDM in two or more pregnancies. Despite differing testing techniques and varying criteria for the diagnosis of GDM, follow-up studies from across the world consistently showed a higher rate of subsequent diabetes among GDM mothers, associated with increased morbidity, and a higher mortality rate. Costs associated with the health of the mother in later years were not considered in the current model and recent epidemiologic data suggest that the real costs of macrosomic birth are considerable higher than presented in this manuscript.

### Long Term Risks of Macrosomia

Fetal macrosomia is a risk factor for the development of obesity in childhood. In the European cohort IDEFICS, children who were macrosomic at birth showed significantly higher actual values of BMI, waist circumference, and sum of skin fold thickness (Sparano et al., 2013).

A recent prospective study, conducted in China, examined the risk factors and long-term health consequences of macrosomia (Gu et al., 2012). Using a population sample of 21 315 mother-child pairs, the children were prospectively followed and assessed for obesity 7 years after birth. Macrosomic infants showed an increased susceptibility to develop childhood overweight and/or obesity. Obesity among children is a significant risk factor for the development of insulin resistance, and the degree of obesity is correlated with the degree of insulin resistance (Arslanian and Suprasongsin, 1996; Young-Hyman et al., 2001). A recent literature review indicates an extra lifetime medical cost of $19,000 for the obese child compared to a normal weight child, in the USA. To put this into perspective, if multiplied with the number of obese 10-year-olds today this yields a total direct medical cost of obesity of roughly $14 billion for this age alone (Finkelstein et al., 2014).

To investigate the relationship between birth weight and later development of GDM, a retrospective study on the medical records of 388 women from Malta, diagnosed for GDM (Savona-Ventura and Chircop, 2003) demonstrated that high birth weight is an important correlate for the subsequent development of GDM in later life. This study further supports the notion that the intrauterine influences on pancreatic development and peripheral response to insulin contribute to the development of adult-onset of T2DM.

Boney examined the development of metabolic syndrome among LGA and appropriate-for-gestational age children (Boney et al., 2005). They observed that obesity among 11-years-old children was a strong predictor for insulin resistance, and the combination of LGA status and a mother with GDM might increase this risk. They also reported that LGA offspring of diabetic mothers were at significant risk of developing metabolic syndrome in childhood.

Again, costs associated with the health of the offspring in later years were not considered in the current model and the above mentioned observations further support the notion that the real costs of macrosomic birth are considerably higher than the outcomes presented in this pilot analysis.

## Conclusion

The health economic decision tree as reported in this paper, allows mapping the short-term care burden and public health impact of complications resulting from GDM and overweight pregnancies. This model gives an impulse for further assessment of the cost-effectiveness of preventive interventions. In addition, as the incidence of macrosomia and related risk-factors will be a key driver for future health care costs, exploration of the most appropriate data sources and assumptions, as well as additional data obtained from longitudinal studies and other epidemiologic recordings, are required to evaluate the long-term consequences.

The current budget impact analysis, using available USA data and on short term costs only, shows that the annual budget impact of GDM and pregnancy overweight resulting in macrosomic birth can be substantial, thus emphasizing the importance of avoiding these adverse health outcomes.

The reported differences on GDM incidence, obesity or the combination thereof, as well as the predisposition in people of Asian ethnicity to develop diabetes and the high proportion of undiagnosed diabetic conditions in this part of the world, stresses the need to collect more country-specific data for improving the assessments of the health economic burden of macrosomic birth and of its later consequences.

The difficulties to change lifestyle and dietary behavior are generally recognized, however, the (pre) pregnancy period offers a window of opportunity for healthcare monitoring and nutritional and lifestyle interventions in the receptive population of future parents. Well-targeted educational programs on lifestyle and food behavior during (pre) pregnancy are likely to improve adverse birth outcomes related to macrosomia. On the long run, this might represent a valuable contribution to the global efforts in the fight against NCD.

## Conflict of Interest Statement

None of the authors have a competing financial interest in relation to the work described; Irene Lenoir-Wijnkoop is employed by Groupe Danone in France, Eline vand der Beek, and Johan Garssen are employed by Nutricia Research in Singapore and the Netherlands, respectively.
